# CLA^+^ T Cell Response to Microbes in Psoriasis

**DOI:** 10.3389/fimmu.2018.01488

**Published:** 2018-06-27

**Authors:** Carmen De Jesús-Gil, Ester Ruiz-Romeu, Marta Ferran, Anca Chiriac, Gustavo Deza, Péter Hóllo, Antonio Celada, Ramon M. Pujol, Luis F. Santamaria-Babí

**Affiliations:** ^1^Translational Immunology, Department of Cellular Biology, Physiology and Immunology, Faculty of Biology, Universitat de Barcelona, Barcelona, Spain; ^2^Department of Dermatology, Hospital del Mar.Barcelona, Barcelona, Spain; ^3^IMIM (Hospital del Mar Medical Research Institute), Barcelona, Spain; ^4^Nicolina Medical Center, Department of Dermatology, Iasi, Romania; ^5^Apollonia University, Iasi, Romania; ^6^“P.Poni” Institute of Macromolecular Chemistry, Romanian Academy, Iasi, Romania; ^7^Department of Dermatovenerology and Oncodermatology, Semmelweis Egyetem, Budapest, Hungary; ^8^Macrophage Biology, Department of Cellular Biology, Physiology and Immunology, Faculty of Biology, Universitat de Barcelona, Barcelona, Spain

**Keywords:** psoriasis, cutaneous lymphocyte-associated antigen, homing, *Candida albicans*, *Streptococcus pyogenes*

## Abstract

*Streptococcus pyogenes* throat infection is a clinically relevant trigger of both guttate and chronic plaque psoriasis, and it provides an ideal context in which to study the pathogenesis of these diseases using an antigen-dependent approach. Circulating cutaneous lymphocyte-associated antigen (CLA) positive (+) memory T cells are a subset of peripheral lymphocytes whose phenotype and function are related to immunological mechanisms in the skin. These cells are considered peripheral biomarkers of T-cell-mediated skin diseases. The coculture of autologous epidermal cells with CLA^+^ T cells from psoriasis patients activated by *S. pyogenes* allows the reproduction of the *ex vivo* initial molecular events that occur during psoriatic lesion formation. With cooperation of autologous epidermal cells, *S. pyogenes* selectively activates CLA^+^ T cells both in guttate and plaque psoriasis, inducing key mediators, including an IL-17 response. Here, we explore potential new mechanisms of psoriasis development including the influence of HLA-Cw6 on *S. pyogenes* CLA^+^ T cell activation in guttate psoriasis, the relevance of IL-9 on microbe induced IL-17 response in guttate and plaque psoriasis, and novel effector functions of *Candida albicans*. This review will summarize recent knowledge of psoriatic mechanisms elicited by microbes that have been studied through an innovative translational perspective based on CLA^+^ T cell-mediated cutaneous immune response.

## Introduction

Molecular studies of psoriasis lesions and patients have allowed translational research to generate potent and novel therapies (1). However, our understanding of the influence of environmental factors on the psoriatic cutaneous immune response is still limited (1). Several microorganisms, including bacteria, fungi, and viruses, have been postulated to be potential triggers and/or exacerbating factors of psoriasis (2). Bacterial genome DNA sequencing in psoriasis is an area of great interest, some microorganisms have been identified but their functional relevance for psoriasis is still to be determined. Psoriasis can be classified as early or late onset (3). The former is associated with the HLA-Cw6 allele, streptococcal throat infection, and a higher tendency to be generalized (4, 5). Interestingly, patients with this type often present a more intense inflammatory lymphocytic infiltrate and are more likely to receive biological therapy (6). All these observations suggest that the presentation of psoriasis is associated with the present bacterial infection. *Streptococcus pyogenes* throat infection is a well-characterized infectious trigger of gutatte psoriasis (GP) and chronic plaque psoriasis (CPP). More than 60 years ago, it was reported that two-third of GP patients present an acute sore throat 12 weeks before the skin eruption (7). Similarly, CPP can also be triggered by *S. pyogenes* throat infections (8), and interestingly, CPP patients have a higher incidence of recurrent sore throats compared with controls (9, 10). The presence of *S. pyogenes* has been detected in the blood of both GP and CPP patients (11). In addition, tonsillectomy can be a useful therapeutic intervention in CPP patients with a history of streptococcal-associated exacerbations (12). It has been proposed that psoriasis tonsillar CLA^+^ T cells (13) activated by streptococcal antigens migrate to the skin where they react to antigens that share sequence homology with the streptococcal proteins (14). However, other microbes may also participate in psoriasis. Fungal cutaneous infections caused by *Candida albicans* have been associated with exacerbation of the disease and a higher frequency of intestinal *C. albicans* isolation in psoriasis patients than controls has been reported (2), although the mechanisms involved in *C. albicans*-induced psoriasis remain poorly characterized.

Interestingly, microbes such as *C. albicans* induce type I interferon response and, type I interferon production by plasmacytoid dendritic cells in skin has been stated to be an important trigger for psoriasis development (15). However, clinical efficacy blocking antibodies against IFN-α have not shown clinical efficacy in psoriasis (16), rising questions about the translational relevance of this mechanism.

In this review, we cover the current state of the art in psoriasis immunopathogenic mechanisms brought out by disease-related microorganisms, such as *S. pyogenes* or *C. albicans*. We focus on cutaneous immune response mediated by CLA^+^ T cells and how these microbes affect T cell activation and production of clinically relevant cytokines.

## Circulating CLA^+^ T Cells and the Study of the Cutaneous Immune System

The immune responses of T cells during cutaneous chronic inflammation in psoriasis involve a subset of memory T lymphocytes that can be distinguished from other memory T cells by the surface expression of the cutaneous lymphocyte-associated antigen (CLA) antigen. This antigen is a cell surface carbohydrate that allows the identification of memory T cells that belong to the cutaneous immune system. CLA is an adhesion molecule expressed by 15% of circulating T cells in humans, and by most (>90%) skin-infiltrating T cells, contrary to other inflamed organs (17). In addition to several ligands for chemokine receptors (CCR10, CCR4, CCR6, and CCR8), CLA binds to E-selectin and together with the interaction between the very late antigen-4/vascular cell adhesion protein-1 and lymphocyte function-associated antigen-1/intercellular adhesion molecule-1, forms a code bar system enabling skin lymphoid infiltration (18). The relevance of circulating CLA^+^ T cells in the cutaneous immune response lies not only in the skin-seeking capacity of these cells but also in their functional relation to the immune response that occurs in inflamed cutaneous lesions. This feature is derived from the recirculating capacity of these cells between skin lesion and blood during cutaneous inflammation (18, 19). The antigen-specific response and phenotype of circulating CLA^+^ T cells has been studied in many human skin conditions. CLA^+^ T cells respond to antigens, allergens, or superantigens involved in disease by triggering T cell-mediated skin diseases, such as psoriasis, atopic dermatitis, and contact dermatitis (18). Furthermore, the phenotype and function of these cells are related to the clinical status of the patient, thereby explaining why circulating CLA^+^ T cells are considered peripheral cell biomarkers of T cell-mediated cutaneous disease in humans (18). Using CLA^+^ T cells from psoriasis patients and healthy controls, our group explores the influence of microbes on cutaneous immune response in psoriasis.

## CLA^+^ T Cell Activation by *S. pyogenes* in Psoriasis Induces IL-17 and IL-9 Responses

Studying the antigen-specific immune response of CLA^+^ T cells induced by clinically relevant triggers of psoriasis may allow the identification of the translational mechanisms involved in psoriasis. The stimulation of autologous coculture CLA^+^ T cells and epidermal cells with *S. pyogenes* leads to an inflammatory immune response that shows the hallmarks of psoriasis. By contrast, the same stimulation of CLA^−^ cells from the same patient or cultures using CLA^+^/CLA^−^ T cells from healthy controls does not have this effect (20). The CLA^+^ T cell response in this model is related to the clinical response of patients in terms of anti-streptolysin O levels, PASI, and duration of flare in GP (21), and to anti-streptolysin O in CPP (20). This activation is determined by the presence of autologous epidermal cells (lesional/non-lesional) and MHC class I and class II presentation. Supernatants of *S. pyogenes*-activated cocultures of CLA^+^ T cells and epidermal cells induce epidermal hyperplasia upon intradermal injection in mouse skin (20). IL-17A and IL-17F production is probably the most relevant effect of *S. pyogenes* on CLA^+^ T cells in psoriasis. The influence of *S. pyogenes* through the response of these cells and the relevance of IL-17 production in GP have been extensively studied (21). In HLA-Cw6^+^ patients whose GP flare is associated with a pharyngitis episode, the Th17-associated response is greater than that exerted by samples from GP patients not associated with pharyngitis. In fact, significantly higher levels of IL-17A, IL-17F, and even IL-6, which participates in Th17-differentiation, were found (21). Thus, the observed response of psoriasis memory T cells to *S. pyogenes* seems to be restricted to CLA^+^ T cells, leading to IL-17 production. This cytokine is a key driver of psoriasis, and its neutralization in patients, or receptor blockade improves the skin condition (1).

The cytokine IL-9 is involved in chronic inflammation and has recently been associated with psoriasis (22, 23). We have demonstrated how *S. pyogenes* preferentially induces IL-9 production during the coculture of autologous CLA^+^ T cells and epidermal cells in psoriasis but not in healthy controls. IL-9 is produced in the same culture conditions in which IL-17A and IL-17F are detected in a time-dependent manner. IL-9 production is dependent on MHC class I and class II presentation, and it preferentially prolongs CLA^+^ T cell survival. Higher amounts of IL-9 were detected in psoriasis patients than in healthy controls, but no differences were observed between GP and CPP patients (24). IL-9 has been associated with increased IL-17A production in an animal model of psoriasis (23). Since *S. pyogenes* induces both IL-9 and IL-17A, we examined the interaction between these two cytokines in CLA^+^ T cells. To this end, we blocked IL-9 function using a neutralizing. A 50% reduction in IL-17A production, but not IFN-γ, was found when was IL-9 was neutralized in CLA^+^ cells activated by *S. pyogenes*.

Our studies have shown that, in CPP patients without clinical evidence of *S. pyogenes* infection, only CLA^+^ T cells respond to this microbe in comparison to healthy controls. This observation indicates that psoriasis patients present an adaptive immune response to *S. pyogenes* through IL-17A, IL-17F, IL-9, and IFN-γ production (20, 21, 24) and suggests that *S. pyogenes* modulates the response of the CLA^+^ T cells that maintain psoriatic lesions, i.e., pyogenes infection has been describe to participate in CPP infection, since higher levels of IgG against *S. pyogenes* proteins are detected in psoriasis patients in comparison to healthy controls (25). Some studies have reported the presence of the genera *Streptococcus* in normal and psoriatic skin (26) and the isolation of *S. pyogenes* in the skin of GP patients (4), probably leading to cutaneous immunization and a CLA^+^ T cell-restricted response in psoriasis.

## CLA^+^ T Cell Response to *C. albicans* in Psoriasis

The cutaneous adaptive immune response to *C. albicans* infection is mediated by a Th17 profile since Th17 cells are essential for anti-fungal barrier immunity (27). Patients with Th17 deficiencies have an increased susceptibility to candidiasis (28), and CD45RA^+^ human T cells may lead to an increase in the number of IL-17 and IFN-γ-producing cells (29). Cutaneous candidal infections have been reported in association with psoriasis exacerbation (30); however, the mechanisms by which *C. albicans* induces psoriasis are poorly understood (2). In psoriasis, *C. albicans*-derived superantigens may induce an expansion of lymphocytes expressing the T-cell receptor variable region beta 5.1 (31). Like in the case of *S. pyogenes*, CLA^+^ T cells, together with autologous epidermal cells, preferentially respond to *C. albicans* extract by inducing IL-9, IL-17A, and IFN-γ production in psoriasis. This response appears to be restricted to CLA^+^CD4^+^ memory T cells since CD4-depleted CLA^+^ memory T cells do not respond to this microorganism in a coculture model with psoriasis cells (24). These results are in line with the expected immune response to *C. albicans* in the skin. However, the observed preferential response of CLA^+^ T cells in psoriasis suggests an adaptive immune response to *C. albicans*, underlying its importance as a relevant antigen likely to be involved in triggering the disease.

## Influence of *S. pyogenes* and *C. albicans* on IL-17 Adaptive Immune Response in Psoriasis

The precise mechanisms by which environmental factors trigger psoriasis are not well understood (1). Biological therapies have revealed the clinical relevance of the IL-23/IL-17 axis in this skin disease. Thus, environmental factors that contribute to fueling the IL-23/IL-17 response may induce the condition. The observation that CLA^+^ T cells in psoriasis patients respond to skin *S. pyogenes* and *C. albicans* extracts indicates a relationship between memory T cells and environmental microbes. Such preferential sensitization to these microorganisms in psoriasis can be either at the tonsillar level in psoriasis through the abnormal generation of CLA^+^ T cells or at the skin level, since the presence of both *S. pyogenes* and *C. albicans* in psoriatic lesions (13). The CLA^+^ T cell response to these microbes is based on IL-17A, IL-17F, IL-9, and IFN-γ production. This response indicates that these skin-homing cells will migrate to psoriatic lesions and thus that they may be involved in the local inflammatory response. IL-17A and IL-17F are clinically validated mediators of psoriasis. *S. pyogenes*-driven IL-9 production through CLA^+^ T cells supports IL-17A production in human lymphocytes, since *in vitro* neutralization of IL-9 reduces IL-17A production by 50% (24).

A current model of IL-17 production in psoriasis considers that some autoantigens, such as LL-37 and ADAMTS-like protein 5, would activate T17 cells (32), initiating the immune circuit of the psoriasis pathogenetic mechanism in the disease. Also, IL-23 production by inflammatory dendritic cells favors the generation and maintenance of the T17 phenotype in psoriasis. Interestingly, regarding the possible interplay between *C. albicans* and *S. pyogenes* and the IL-23/Th17 axis, it has been recently shown that *C. albicans* stimulates dendritic cells to release IL-23 (33). There is a complex interplay between these two microbes and CLA^+^ T cells in psoriasis; however, the influence of microbes in psoriasis may be more complex that originally believed since microbiota studies demonstrate the presence of a range of microorganisms in psoriatic lesions (34). The functional relevance of these microorganisms for the disease has not been determined (35).

**Figure 1:**
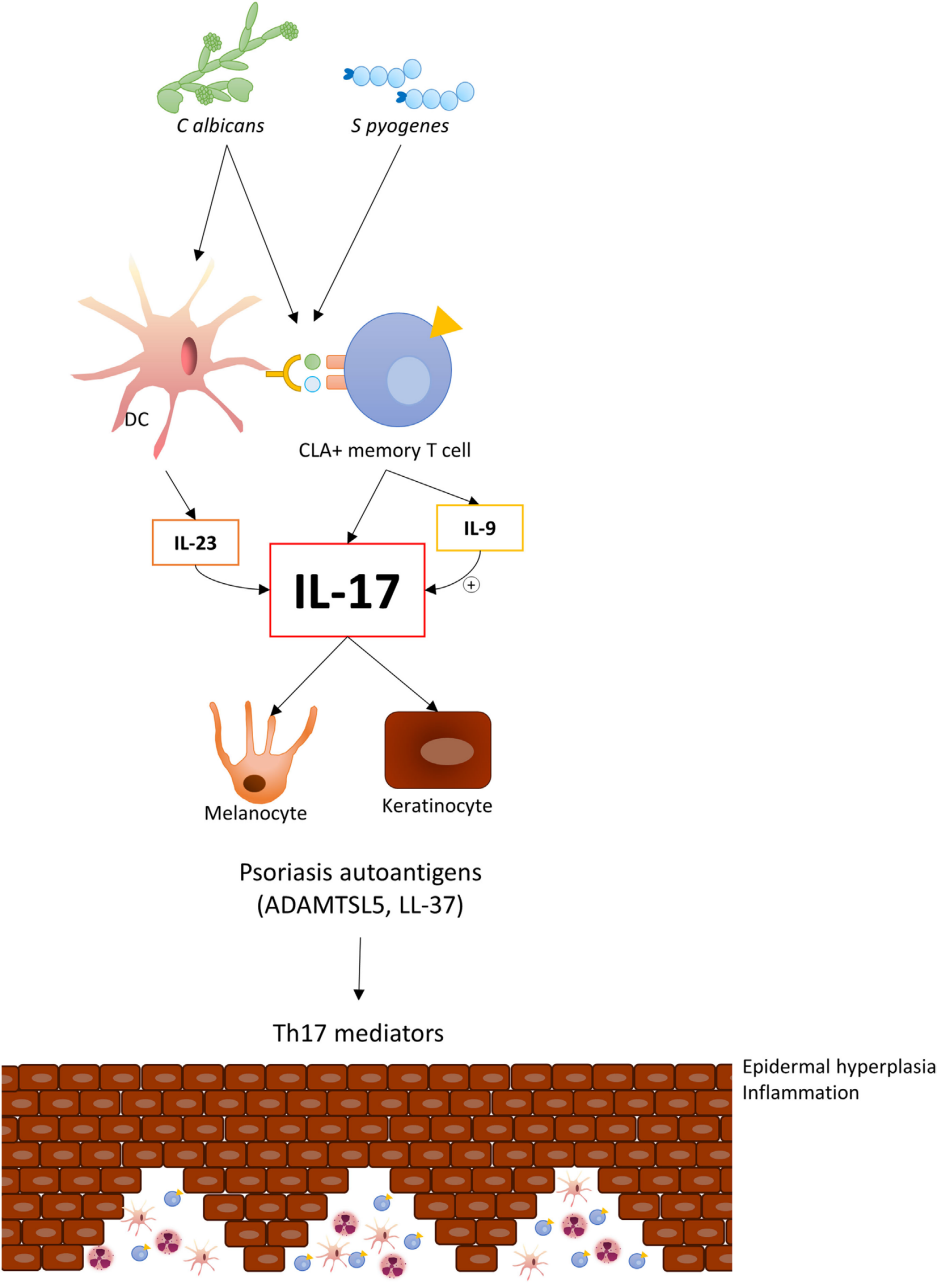
*Candida albicans* and *Streptococcus pyogenes* induce IL-17 response in circulating CLA^+^ T cells in psoriasis, thereby indicating an established adaptive immune response to these microorganisms in this disease. Upon migration to cutaneous lesions, these cells react with those microbes and locally trigger IL-17 and IL-9 production, which will contribute to inducing psoriasis autoantigens.

In summary, the observations made to date suggest that circulating CLA^+^ T cells in psoriasis patients produce increased amounts of IL-17A, IL-17F, and IL-9, in comparison to healthy controls, when activated by *S. pyogenes* (Figure 1). Interestingly, the response to *C. albicans* is restricted mainly to CLA^+^ T cells in cocultures with autologous epidermal cells in psoriasis with a similar cytokine profile. Psoriatic lesions produce several chemokines to attract skin-seeking CLA^+^ T cells (21) with IL-17 capacity to the skin with potential to induce IL-17-dependent autoantigens and promote and maintain lesion activity. The study of the cutaneous immune response of CLA^+^ T cells allows us to gain insight into how environmental factors, such microbes, shape psoriasis inflammation.

## Author Contributions

All authors listed have made a substantial, direct, and intellectual contribution to the work and approved it for publication. LFSB conceived the ideas and together drafted the manuscript. All authors revised and approved the final version of the manuscript.

## Conflict of Interest Statement

The authors declare that the research was conducted in the absence of any commercial or financial relationships that could be construed as a potential conflict of interest.
